# Drivers of diversification in *Linum* (Linaceae) by means of chromosome evolution: correlations with biogeography, breeding system and habit

**DOI:** 10.1093/aob/mcad139

**Published:** 2023-09-20

**Authors:** Ana Valdés-Florido, Lu Tan, Enrique Maguilla, Violeta I Simón-Porcar, Yong-Hong Zhou, Juan Arroyo, Marcial Escudero

**Affiliations:** Departamento de Biología Vegetal y Ecología, Facultad de Biología, Universidad de Sevilla, Avenida Reina Mercedes no. 6, 41012, Seville, Spain; Panxi Crops Research and Utilization Key Laboratory of Sichuan Province, Xichang University, Xichang, Sichuan, 615000, China; Triticeae Research Institute, Sichuan Agricultural University, Chengdu, Sichuan 611130, China; Departamento de Biología Vegetal y Ecología, Facultad de Biología, Universidad de Sevilla, Avenida Reina Mercedes no. 6, 41012, Seville, Spain; Área de Botánica, Departamento de Biología Molecular e Ingeniería Bioquímica, Universidad Pablo de Olavide, Ctra de Utrera km 1 sn, 41013, Seville, Spain; Departamento de Biología Vegetal y Ecología, Facultad de Biología, Universidad de Sevilla, Avenida Reina Mercedes no. 6, 41012, Seville, Spain; Triticeae Research Institute, Sichuan Agricultural University, Chengdu, Sichuan 611130, China; Departamento de Biología Vegetal y Ecología, Facultad de Biología, Universidad de Sevilla, Avenida Reina Mercedes no. 6, 41012, Seville, Spain; Departamento de Biología Vegetal y Ecología, Facultad de Biología, Universidad de Sevilla, Avenida Reina Mercedes no. 6, 41012, Seville, Spain

**Keywords:** Breeding system, diversification, dysploidy, flax, heterostyly, Linaceae, polyploidy

## Abstract

**Background and Aims:**

Chromosome evolution leads to hybrid dysfunction and recombination patterns and has thus been proposed as a major driver of diversification in all branches of the tree of life, including flowering plants. In this study we used the genus *Linum* (flax species) to evaluate the effects of chromosomal evolution on diversification rates and on traits that are important for sexual reproduction. *Linum* is a useful study group because it has considerable reproductive polymorphism (heterostyly) and chromosomal variation (*n* = 6–36) and a complex pattern of biogeographical distribution.

**Methods:**

We tested several traditional hypotheses of chromosomal evolution. We analysed changes in chromosome number across the phylogenetic tree (ChromEvol model) in combination with diversification rates (ChromoSSE model), biogeographical distribution, heterostyly and habit (ChromePlus model).

**Key Results:**

Chromosome number evolved across the *Linum* phylogeny from an estimated ancestral chromosome number of *n* = 9. While there were few apparent incidences of cladogenesis through chromosome evolution, we inferred up to five chromosomal speciation events. Chromosome evolution was not related to heterostyly but did show significant relationships with habit and geographical range. Polyploidy was negatively correlated with perennial habit, as expected from the relative commonness of perennial woodiness and absence of perennial clonality in the genus. The colonization of new areas was linked to genome rearrangements (polyploidy and dysploidy), which could be associated with speciation events during the colonization process.

**Conclusions:**

Chromosome evolution is a key trait in some clades of the *Linum* phylogeny. Chromosome evolution directly impacts speciation and indirectly influences biogeographical processes and important plant traits.

## INTRODUCTION

Variation in chromosome number resulting from the balance between polyploidy (whole genome duplication, WGD) and dysploidy (changes in chromosomal number without variation of ploidy levels) is considered a key driving force in the speciation and diversification of plants (Stebbins, 1950; Grant, 1981; Soltis *et al.*, 2009, 2015; Landis *et al.*, 2018). Chromosomal rearrangements are often associated with species differentiation (White, 1978), as they have the potential to reduce gene flow between diverging populations (Grant, 1981). There are two alternative general models of the role of chromosomal evolution in species divergence (Ayala and Coluzzi, 2005). The ‘hybrid-dysfunction’ model presumes reduced fitness of hybrids between chromosome races. Meanwhile, the ‘suppressed recombination’ model assumes that chromosome rearrangements act as genetic filters between populations, since mutations appearing in the newly unpaired chromosomes cannot flow between populations. The ‘suppressed recombination’ model has stronger theoretical support, since the ‘hybrid-dysfunction’ model requires the unlikely process of a single individual with the new chromosome rearrangement being established in the population (Ayala and Coluzzi, 2005). Apart from its direct role in speciation, chromosome evolution also seems to play a reinforcing role in the speciation process (de Vos *et al*., 2020).

The role of WGD on plant diversification rates is controversial (Soltis *et al*., 2014; Mayrose *et al*., 2015). Recently formed polyploid plants have been suggested to diversify at lower rates, since polyploid events are usually detected towards the tips of phylogenies (Mayrose *et al*., 2011). However, many plant radiations appear to have been preceded by polyploid events (Soltis *et al*., 2009). Furthermore, a more recent study concluded that there is no significant association at all between shifts in diversification rates and ancient WGDs in angiosperms (Landis *et al*., 2018). These apparent inconsistencies could be at least partly explained by the recent recognition that post-polyploidy diploidization processes and chromosomal fusion may confound inferences of WGD based on chromosome numbers alone (Wendel, 2015; Escudero and Wendel, 2020). Knowledge about the relationship between dysploidy and diversification rates is limited, even though dysploidy persists longer over evolutionary time than polyploidy (Escudero *et al*., 2014).

A variety of plant traits have been related to chromosome evolution, and specifically to polyploidy. Among reproductive traits, both clonality (Herben *et al.*, 2017) and self-fertilization (Stebbins, 1950; Cook and Soltis, 2000; Tate and Simpson, 2004; Barringer, 2007) have been suggested to be positively associated with polyploidy. However, reaching general conclusions for both traits has so far proved difficult (Mable, 2004; Van Drunen and Husband, 2019). Earlier analyses (Mable, 2004) at wide taxonomic scales failed to detect a relationship between polyploidy and self-compatibility (SC). However, it is important to account for phylogenetic effects in the data set, and also to recognize that SC is not exactly the same as selfing, which is the expected selected outcome after sexual selection processes (Lloyd, 1992; Cutter, 2019).

The relationship between ploidy level, SC (and consequently a higher selfing rate) and diversification patterns has been recently inferred for the first time in the genus *Solanum* (Zenil-Ferguson *et al*., 2019). The relationships between these three variables were complex, with diversification being related to SC and another unobserved factor, but not to ploidy level, under the assumption that polyploidy is directly linked to SC. The positive association between polyploidy and selfing could be explained by three non-mutually exclusive hypotheses: (1) by the effect of WGD masking inbreeding depression, which would facilitate the transition to selfing (Barringer, 2007; Barrett, 2008; Husband *et al*., 2008); (2) because self-fertilization may facilitate the establishment of polyploids by avoiding the lower fitness of triploids (Ramsey & Schemske, 1998; Husband *et al*., 2008); and (3) in RNase-based gametophytic self-incompatibility (SI) systems, polyploidization might directly cause the loss of SI alleles (Stout & Chandler, 1942; Lewis, 1947; Vieira *et al.*, 2008). If we assume that polyploidy is positively associated with selfing, it can be inferred that breeding systems promoting outcrossing should be negatively associated with polyploidy. This might apply for mechanisms that strongly facilitate outbreeding, such as SI (Stebbins, 1950; Cook and Soltis, 2000), gender separation (dioecy and related conditions; Pannell *et al*., 2004; Ashman *et al*., 2013), sex organ separation (dichogamy and herkogamy, but see Mable, 2004) and reciprocal style-length polymorphisms (heterostyly and related polymorphisms; Naiki, 2012; de Vos *et al*., 2014*a*). Heterostyly is a stylar polymorphism in which a single species or population contains different floral morphs whose styles and stamens have different and reciprocal heights. The correlation between polyploidization and the loss of heterostyly has already been demonstrated in two major heterostylous families: Primulaceae (Guggisberg *et al*., 2006; Naiki, 2012) and Rubiaceae (Nakamura *et al.*, 2007; Naiki, 2012). Polyploid taxa in those families tend to show derived monomorphic homostylous (i.e. non-herkogamous) flowers and, in some cases, they are frequently selfers due to the breakdown of the distyly supergene (Barrett and Shore, 1987; de Vos *et al*., 2014*b*).

The impact of chromosome evolution on the biogeographical setting of species is evident from the uneven distribution of polyploids, which increase in frequency with increasing latitude (Stebbins, 1971; Rice *et al*., 2019). This pattern could also be explained by several non-exclusive hypotheses. The frequency of unreduced gametes (i.e. gametes with somatic chromosome numbers resulting from failed meiosis) may increase at higher latitudes as the result of harsh and fluctuating (i.e. stressing) environments (Ramsey and Schemske, 1998). At the same time, polyploids might colonize those environments more easily because their fixed heterogenous genomes reduce the effects of inbreeding depression and genetic drift during climate changes (Brochmann *et al*., 2004). (Allo)polyploids have also been suggested to be more abundant in recently deglaciated regions as allopatric populations were brought into contact following ice retreat (Stebbins, 1984). Life history traits could mediate the geographical pattern of polyploid distribution, as polyploids are disproportionately more frequent among perennial herbs, which are more likely to have vegetative propagation and are relatively more frequent at higher latitudes than annual species (Stebbins, 1971). Finally, polyploids have been hypothesized to have higher adaptability and evolutionary potential than diploids because of the high genetic diversity granted by the polyploidization event itself (Soltis *et al.*, 2011; Tank *et al.*, 2015; Van der Peer *et al.*, 2017), which also confers higher plasticity (Te Beest *et al.*, 2012). It is noteworthy that taxonomy may bias our knowledge of polyploid distribution (Rice *et al*., 2019), as some taxonomists tend to treat polyploids as distinct species, while others consider variation in chromosome number as taxonomically unimportant, leading to an underestimation of the number of polyploid species.

The sub-cosmopolitan genus *Linum* (Linacee) has high species-richness in different regions of the world, high variability in the reproductive traits of style length polymorphism and incompatibility system (Dulberger, 1992), and complex biogeographical patterns (Maguilla *et al*., 2021). Although there is not enough direct empirical data on SI systems of *Linum* species, there is substantial knowledge on the distribution of heterostyly in the genus (Ruiz-Martín *et al.,* 2018), in which it is tightly associated with the so-called heteromorphic SI system (Murray, 1986; Dulberger, 1992). Thus, heterostyly is a reasonable proxy for SI in *Linum*. This genus also has wide variation in chromosome number – from *n* = 6 to *n* = 36 – being not homogeneous across all clades, suggesting that both polyploidy and dysploidy may have been important in its evolution (Nicholls, 1986; Bolsheva *et al*., 2017; Afonso *et al*., 2021). Specifically, genomic analyses in *Linum usitatissimum* (Wang *et al*., 2012) showed events of whole genome duplications around 5–9 Mya, and Sveinsson *et al*. (2014) suggested that similar events occurred in Sections *Linum* and *Dasylinum* around 23–42 Mya, showing the importance of these events in the genus. The ancestral chromosome number in *Linum* has not yet been inferred, although it has been estimated as *n* = 6 for the family Linaceae (Raven, 1975; Carta *et al.,* 2020).

Hence, *Linum* provides an excellent opportunity to investigate how chromosome evolution, life form, reproductive traits and biogeography could be correlated, shaping its diversification patterns. In a previous approach, Ruiz-Martín *et al.* (2018) found little evidence of phylogenetic correlation between life history (annual vs. perennial), polyploidy (diploids vs. polyploids) and heterostyly [monomorphism (i.e. with only one floral morph within each population) vs. polymorphism (i.e. with two or more floral morphs within each population)] in the genus. In another recent study that integrated the biogeographical component, Maguilla *et al*. (2021) found that the Western Palearctic acted as a main source of dispersal events in the genus. Interestingly, all of the species or lineages that colonized new areas after long-distance dispersal were style-monomorphic (which are more likely to be selfers than heterostylous species); this is consistent with the theoretical expectations of reproductive traits among colonizing species laid out in Baker’s law (Baker, 1974). However, neither biogeographical changes nor breeding system changes could explain speciation or extinction rates in *Linum* in the study of Maguilla *et al*. (2021). Despite chromosome number being quite variable in *Linum*, its evolution remains poorly studied (but see Sveinsson *et al.*, 2014), leaving practically unknown the role of chromosome number evolution in shaping the relationship of the above traits and diversification rates in the genus. Finally, recent genomic analyses in the genus (Gutiérrez-Valencia *et al*., 2022) revealed the significance of genetic architecture in the evolution of stylar polymorphism. This architecture might be related to changes in chromosome evolution across the genus and may thus be favoured under certain ecological conditions, such as harsh abiotic factors or lack of pollinators (Rifkin *et al.*, 2021).

Here, we aimed to analyse variations in chromosome number across the *Linum* phylogeny using modern tools to determine the effect of chromosome number variation on the diversification rates of the genus and to infer whether chromosome evolution plays a role in (1) reproductive traits (heterostyly), (2) the biogeography of the genus (species within the original area of the genus vs. species in newly colonized areas) and (3) species habit (annual vs. perennial). We hypothesize that chromosome evolution played a role in cladogenetic processes in *Linum*. We also expect polyploidy to be negatively associated with heterostyly and positively associated with newly colonized regions and perennial life-form.

## MATERIAL AND METHODS

### Study group and data

For our analyses, we utilized the dated phylogenetic reconstruction published by Maguilla *et al*. (2021), excluding tips for which we did not have information on chromosome number. This reconstruction was made using concatenated sequences of the nuclear ITS and plastid DNA regions *ndh*F, *mat*K and *trn*L-F, downloaded from NCBI GenBank. The phylogenetic analyses were conducted in BEAST 2.4.0 (Bouckaert *et al.*, 2014), using a GTR+I+G model, based on the results of jModelTest 2.1.3 (Darriba *et al*., 2012). In total, our sampling comprised 55 species and one subspecies of *Linum* plus four samples of species of different genera within the monophyletic core *Linum* (hereafter *Linum s.l.*; see Maguilla *et al*., 2021): *Cliococca selaginoides* (Lam.) C.M. Rogers and Mildner, *Hesperolinon micranthum* (A. Gray) Small, *Radiola linoides* Roth. and *Sclerolinon digynum* (A. Gray) C.M. Rogers (Supplementary Data Table S1). The species *Reinwardtia indica* Dumort was included as the outgroup. Chromosome numbers for each species were obtained from the Chromosome Count Database (CCDB; Rice *et al*., 2015), and heterostyly (monomorphic vs. polymorphic) and life history (annual vs. perennial) were coded following Ruiz-Martín *et al*. (2018). We used here the term style monomorphism when only one style morph is reported, and only will refer to homostyly when there is monomorphism in addition to lack of herkogamy.

Finally, we scored each taxon’s occurrence in the ancestral vs. newly colonized areas following the results of the biogeographical reconstruction published by Maguilla *et al*. (2021; Supplementary Data Table S1).

### Chromosome evolution modelling

Phylogenetic chromosome evolution modelling has experienced a revolution in the past decade. The first ChromEvol model accounted for three main events: an increase by a single chromosome number (ascending dysploidy), a decrease by a single chromosome number (descending dysploidy) and duplications of the chromosome number (i.e. WGD or polyploidy) (Mayrose *et al*., 2010). Two additional rate parameters allow the ascending and descending dysploidy rates to depend linearly on the current number of chromosomes, and a third parameter, defined as demiduplication or demipolyploidy, permits multiplications of the number of chromosomes by 1.5.

Subsequently, the ChromEvol 2.0 (Glick and Mayrose, 2014) model implemented two additional parameters: the base number and its respective transition rate by multiplication of the base number. There have also been two recent approaches to jointly model chromosome evolution and binary traits: the BiChrom model (Zenil-Ferguson *et al*., 2017) and the ChromePlus model (Blackmon *et al*., 2019). Both models allow a binary trait to affect the rate of chromosome number change, and the ChromePlus model also allows for the binary trait to affect rates of speciation and extinction, following the BiSSE modelling framework (Maddison *et al*., 2007). Finally, Freyman and Höhna (2018) proposed the ChromoSSE model which allows both anagenetic and cladogenetic chromosome number transitions, effectively linking the process of diversification to chromosome number change.

For this study, we first used the ChromEvol 2.0 model to infer the evolution of chromosome number across the phylogeny (Mayrose *et al*., 2010; Glick and Mayrose, 2014). We tested ten models of chromosome evolution that combine eight different parameters (chromosome gain, chromosome loss, linear chromosome gain, linear chromosome loss, polyploidy, demipolyploidy, base number rate and base number estimation) related to dysploidy and polyploidy were tested (Escudero *et al*., 2023). The analyses were performed following Escudero *et al*. (2014). We used Akaike’s information criterion (AIC) to compare among models and choose the best-fitting model of chromosome evolution, which we later used to reconstruct and plot chromosome numbers across the phylogeny. The plot was made using the ChromEvol functions v.1 of N. Cusimano https://www.en.sysbot.bio.lmu.de/people/employees/cusimano/use_r/) in R. Some *Linum* species display intraspecific chromosome number variation. We therefore repeated the analysis three times: (1) considering within-species variation (i.e. indicating the proportion of each chromosome number); (2) considering only the most probable chromosome number; and (3) considering only the median chromosome number. The last dataset was used for all further analyses.

### Chromosomal cladogenesis

Models implemented in ChromEvol (Mayrose *et al*., 2010; Glick and Mayrose, 2014) assume that chromosomal transitions happen exclusively in the branches (anagenetically), i.e. excluding the possibility that chromosomal transitions result in speciation. In contrast, the ChromoSSE model (Freyman and Höhna, 2018), which has been implemented in the RevBayes platform (Höhna *et al*., 2016), allows for chromosomal transitions to happen both anagenetically and cladogenetically. We thus used this model to test whether cladogenetic events were related to chromosomal transitions. The default model has 13 parameters: root frequencies, relative extinction, six anagenetic parameters (chromosome gain, chromosome loss, linear chromosome gain, linear chromosome loss, polyploidy and demipolyploidy) and five cladogenetic parameters (no chromosomal change, chromosome gain, chromosome loss, polyploidy and demipolyploidy). Based on the ChromEvol results, we simplified the default model by removing the linear gain and loss parameters and constraining the polyploidy and demipolyploidy rates to be equal. This resulted in a model with three anagenetic parameters (chromosome gain, chromosome loss and polyploidy/ demipolyploidy) and four cladogenetic parameters (no change, chromosome gain, chromosome loss and polyploidy/ demipolyploidy).

### Chromosomal evolution and traits

The R package ChromePlus (Blackmon *et al*., 2019) implemented new models for chromosome evolution, including a model with a binary trait that impacts chromosome evolution (a different model of chromosome evolution is inferred for each state of the binary trait). It also implements a more complex model with different rates of chromosome evolution, speciation and extinction rates associated with the binary character [e.g. the BiSSE model (Maddison *et al*., 2007), with a model of chromosome evolution associated with each state of the binary trait]. We discarded the latter model because of its complexity and because two of the traits analysed here – heterostyly (monomorphic vs. polymorphic) and biogeography (ancestral vs. newly colonized areas) – were unrelated to diversification rates in the study by Maguilla *et al*. (2021). Exploratory analyses of the third trait we considered here – life history (annual vs. perennial) – indicated that it is also unrelated to diversification rates. Using the R package Diversitree (FitzJohn, 2012), we compared the AIC of a model of dependent evolution of each of the binary traits and chromosome evolution (parameter transitions – q01 and q10 – and chromosome parameters: chromosome gain – gain0 and gain1, chromosome loss – loss0 and loss1, polyploidy – polyploidy0 and polyploidy1, and demipolyploidy – demipoliplidy0 and demipolyploidy1) against a model of independent evolution of the binary trait and chromosome evolution (parameter transitions – q01 and q10 – and chromosome parameters: chromosome gain, chromosome loss, polyploidy and demipolyploidy).

## RESULTS

### Chromosome evolution

**Fig. 1.**
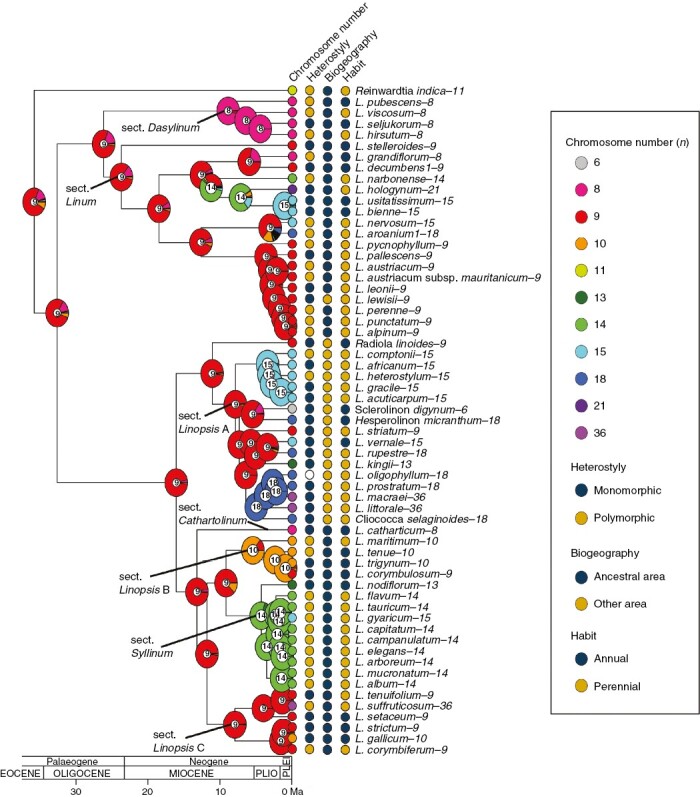
Chromosome number reconstruction based on the ChromEvol ‘CONST_RATE_DEMI’ model for the dataset with the median chromosome number. Chromosome numbers and probabilities (in plot charts) are shown with different colours. Trait states for chromosome number, heterostyly, biogeography and habit are indicated at the tips of the phylogeny.

The inferred models for each of the three different datasets were very similar. The LINEAR_RATE_DEMI model was inferred for the dataset with the most probable chromosome number, and the CONST_RATE_DEMI model was inferred for the datasets with the median chromosome number and considering all chromosome number variation. The two CONST_RATE_DEMI models had a rate of polyploidy equal to the rate of demipolyploidy, a rate of chromosome gain and a rate of chromosome loss, while the LINEAR_RATE_DEMI model had two additional linear rates of chromosome gain and loss. The chromosome number reconstruction was identical under all three methods (Fig. 1 and Supplementary Data Figs S1 and S2).

The ancestral chromosome number of the genus *Linum* was estimated to be *n* = 9. The following chromosomal transitions were inferred at the nodes of the phylogeny: (1) three events of chromosome gains at the origin of clade B of sect. *Linopsis*, at the origin of the South African clade (within sect. Linopsis -clade A-), and in a clade within section *Linum* (the clade that includes *L. bienne* and *L. usitatissimum*); (2) one event of chromosome loss, at the origin of section *Dasylinum*; (3) three demipolyploidy events, at the origin of the section *Syllinum*, at the origin of the South African clade (within sect. Linopsis -clade A-), and in a clade within section *Linum* (the last common ancestor of *L. narbonense* and *L. usitatissimum*); and (4) one polyploid event, at the origin of the South American clade (within sect. *Linopsis* -clade A-). The remaining chromosomal changes (seven events of chromosome gains, eight of chromosome losses, seven polyploid events and four demipolyploid events) were inferred at the tips of the phylogeny.

### Chromosomal cladogenesis

**Fig. 2.**
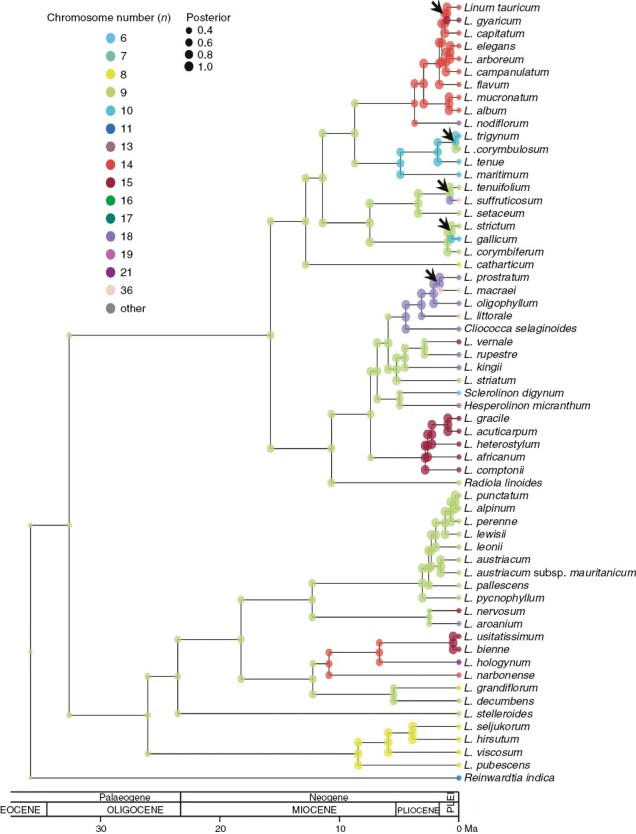
Chromosome number reconstruction based on the ChromoSSE model for the dataset with the median chromosome number. Chromosome numbers are shown with different colours and posterior probabilities with the size of the dots. Chromosomal cladogenetic events are indicated with arrows.

**Fig. 3.**
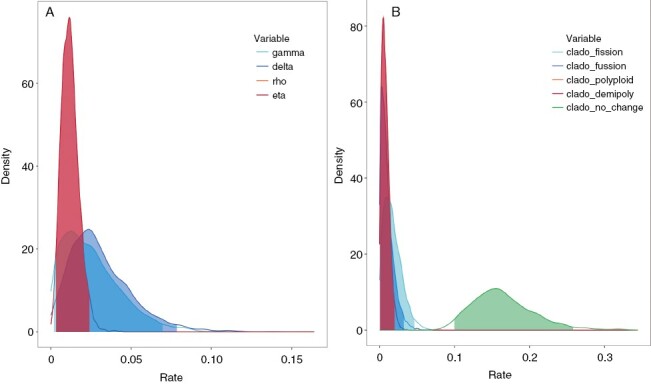
(A) Posterior probability densities of the estimates of the anagenetic parameters in the ChromoSSE model. The *x*-axis displays the rate of anagenetic parameters; the *y*-axis indicates the posterior probability density of each value. (B) Posterior probability densities of the estimates of the cladogenetic parameters in the ChromoSSE model. The *x*-axis displays the rate of cladogenetic parameters; the *y*-axis indicates the posterior probability density of each value.

The ChromoSSE model reconstruction (Fig. 2) was identical to those made using the ChromEvol model (Fig. 1 and Supplementary Data Figs S1 and S2). The posterior distribution of the anagenetic parameters (Table 1; Fig. 3) suggested a slightly higher contribution of anagenetic events compared to cladogenetic events. The most important cladogenetic parameter was that of no-change, which was an order of magnitude higher than parameters of chromosomal cladogenesis (Table 1). Despite this, there were five inferred events of chromosomal speciation. Increasing dysploid chromosomal speciation was detected for *L. gyaricum* and *L. gallicum*. Decreasing dysploid chromosomal speciation was inferred for *L. corymbulosum*. Finally, polyploid speciation was detected for *L. suffruticosum* and *L. macraei*.

**Table 1.** Results from ChromoSSE analyses. Cladogenetic (*clado*), anagenetic (*ana*) and extinction (*extinction*) rate events are shown. The mean, median, 95% confidence interval (*95% CI*) and the explained sum of squares (*ESS*) were calculated for the estimated rates of fission (*fiss*), fusion (*fus*) and no-change (*no-change*), as well as the ratio between polyploidy and demipolyploidy (*poly/demi*) and between demipolyploidy and polyploidy (*demi/poly*).

	clado				ana			extinction
	*poly/demi*	*fiss*	*fus*	*no-change*	*demi/poly*	*fiss*	*fus*	
Mean	0.0081	0.0176	0.0103	0.1644	0.0121	0.0257	0.031	0.4737
Median	0.0072	0.0155	0.0080	0.1592	0.0116	0.0224	0.0272	0.4896
*95% CI*	0.000005–0.0179	0.00001–0.0895	0.000006–0.0276	0.094–0.2466	0.0027–0.0231	0.00006–0.0607	0.00001–0.0677	0.0336–0.8082
*ESS*	2131	2436	2945	1399	1866	879	1150	1191

### Chromosomal evolution, geographical range and traits

**Fig. 4.**
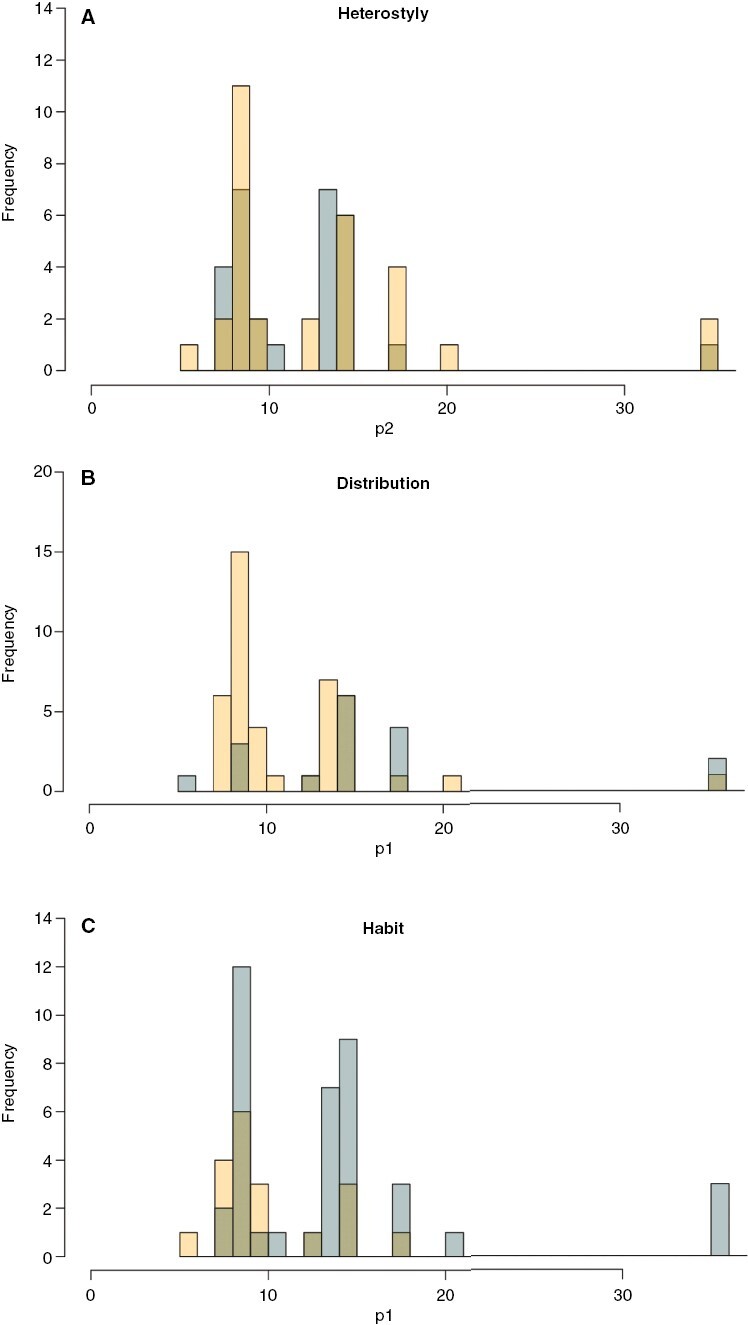
Histograms of chromosome number for (A) heterostyly: monomorphic (yellow) vs. polymorphic (blue); (B) biogeography: source (yellow) vs. colonized (blue) areas; and (C) habit: annual (yellow) vs. perennial (blue). The *x*-axis indicates the chromosome number and the *y*-axis displays the frequency of the chromosome number for each trait.

Chromosome numbers overlapped strongly between stylar polymorphic and monomorphic species (Fig. 4A). Accordingly, the model of independent evolution of heterostyly (monomorphic vs. polymorphic conditions) and chromosome number variation was better supported, while the hypothesis of their correlated evolution was rejected (Table 2).

**Table 2.** Results from ChromePlus analyses. Models of dependent and independent (*Depen Chrom* and *Indepen Chrom*) chromosome changes correlated with heterostyly (*Het*), biogeographical areas (*Biogeo*) and habit (*Habit*). *Df* indicates the calculated degrees of freedom and *LnLik* the log-likelihood. Akaike’s information criterion (*AIC*) was used to choose the best-fitting model of chromosome evolution. Monomorphic *Linum* species were coded as ‘1’ and polymorphic as ‘2’. Species distributed in the original (Palaeoarctic) area were tagged as ‘1’ and those distributed in the colonized areas as ‘2’. Regarding habit, annual species were coded as ‘1’ and perennial as ‘2’. Ascending (*asc1*, *asc2*) and descending (*desc1*, *desc2*) dysploidy, polyploidy (*pol1*, *pol2*) and demipolyploidy (*dem1*, *dem2*) rates were estimated for traits 1 and 2. Transition rates from trait 1 to trait 2 and vice versa were also estimated (*tran12*, *tran21*). The best-fitting model for each studied trait is in bold font.

Model	*Df*	*LnLik*	*AIC*	*asc1*	*asc2*	*desc1*	*desc2*	*pol1*	*pol2*	*dem1*	*dem2*	*tran12*	*tran21*
*Depen Chrom Het*	10	−158.05	336.09	0.05544	0.04719	0.0000009	0.07833	0.0000001	0.05000	0.00508	0.03113	0.12605	0.12463
***Indepen Chrom Het***	**6**	**−161.67**	**335.3**	**0.03922**		**0.03532**		**0.02102**		**0.01844**		**0.18533**	**0.14310**
***Depen Chrom Biogeo***	**10**	**−129.47**	**278.95**	**0.02827**	**0.09425**	**0.00000002**	**0.01863**	**0.07014**	**0.01146**	**0.09605**	**0.01273**	**0.00000004**	**0.00635**
*Indepen Chrom Biogeo*	6	−134.63	281.25	0.03924		0.03533		0.021025		0.01841		0.000002	0.00626
***Depen Chrom Habit***	**10**	**−143.70**	**307.41**	**0.041379**	**0.029618**	**0.0000001**	**0.06798**	**0.03372**	**0.00241**	**0.03645**	**0.00000003**	**0.06951**	**0.07795**
*Indepen Chrom Habit*	6	−150.83	313.65	0.04076		0.03570		0.01875		0.01888		0.077789	0.10780

**Fig. 5.**
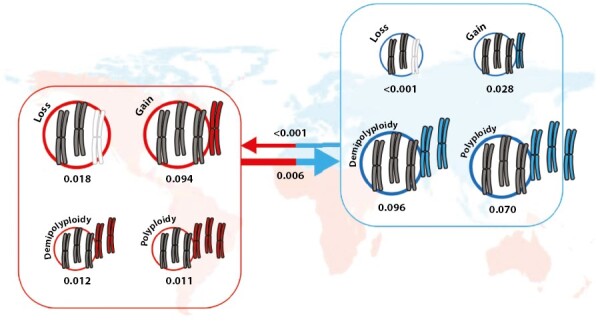
Correlation between chromosome evolution and biogeography. Values indicate the rates of chromosomal change for *Linum* species in the source area (blue) and in colonized (red) areas. Rates are proportional to arrow and circle thicknesses.

Palearctic species were mostly diploid whereas species in the rest of the distribution were mostly polyploids (Fig. 4B). The hypothesis of the correlated evolution of distribution (origin – Palearctic – vs. colonized areas – rest of the distribution) and chromosome number variation was significantly supported (Table 2). The rates of ascending and descending dysploidy were higher in the colonized areas, while the rates of polyploidy and demipolyploidy were higher in the original areas (Fig. 5).

Most of the species were perennial; approximately half of these species were diploids and the other half polyploids (Fig. 4C). Less than one-third of the species were annual, and most of them were diploids (Fig. 4C). The hypothesis of the correlated evolution of habit (annual vs. perennial) and chromosome variation was significantly supported (Table 2). The rates of polyploidy and demipolyploidy were much higher for annual than for perennial species, while the rates of descending dysploidy were much higher for perennial than for annual species. The rates of ascending dysploidy were higher for annual than for perennial species. Although most of the annual species were diploids, there were two events of polyploidization at the tips of the phylogeny (*L. vernale* and *Hesperolinon micranthum*) that probably account for the faster rates of polyploidization associated with annual plants. Among the perennial plants, the inferred polyploidization events occurred at deep nodes in the phylogeny (sect. *Syllinum*, two subclades within sect. *Linopsis* A and a clade of sect. *Linum*) as well as at the tips (*L. suffruticosum*, *L. kingii*, *C. selaginoides*, *L. aroanum*, *L. nervosum* and *L. hologynum*); this may explain the apparently slower rates of polyploidization in the perennials. Chromosome losses were inferred in the polyploid perennial *L. kingii* and also in several diploid species, so such losses cannot be attributed to post-polyploid diploidization events.

## DISCUSSION

### Patterns of chromosome evolution and its effects on diversification rates

Our results suggest an ancestral chromosome number of *n* = 9 for the genus *Linum* (Fig. 1). There are no previous chromosomal reconstructions for this genus, although the ancestral chromosome number for the family Linaceae has long been estimated to be *n* = 6 (Raven, 1975). An ancestral chromosome number of 6 was also the most strongly supported estimate found by Carta *et al.* (2020) using ChromEvol models in global phylogenies of angiosperms. Interestingly, however, the second most likely ancestral chromosome number for Linaceae (with a similar probability) was 9 (Carta *et al.*, 2020), the same as our estimate for *Linum*.

The genus *Linum* shows high rates of chromosomal evolution through both polyploidization and dysploidization events. These events occur mainly at the tips of the phylogeny, but also at some nodes (see Fig. 1). Phylogenetic models across vascular plants have inferred most polyploid events at the tips of the phylogeny and not at the nodes, suggesting limited evolutionary success of polyploidization (Mayrose *et al*., 2011). In fact, recently formed polyploid plants (i.e. neopolyploids) seem to diversify at lower rates than diploids (Mayrose *et al*., 2011). In contrast, the evolutionary success of dysploidy could be higher than polyploidy, as dysploidization events are equally inferred at the tips and in the nodes (Escudero *et al*., 2014).

ChromEvol and similar models that infer patterns of chromosome evolution based on chromosome counts and a phylogeny can be confounded by the post-polyploid diploidization process (genome downsizing and chromosomal fusions; Escudero and Wendel, 2020). In other words, the so-called ‘wondrous cycles of polyploidy in plants’ (Wendel, 2015) may completely obscure the signal of previous polyploidization events. In fact, deep polyploidization events that were known from comparative genomics were not inferred by Carta *et al*. (2020) in the phylogeny of the angiosperms using the ChromEvol model (Escudero and Wendel, 2020). The risk of ignoring previous polyploid events increases as we model chromosome number evolution deeper on a phylogeny. However, this is probably not a significant issue when modelling chromosome evolution at the genus level (see Mayrose *et al*., 2011).

The patterns we inferred in *Linum* suggest that chromosome evolution could drive diversification patterns based on the evolutionary success of all kinds of chromosome number transitions. However, when using more complex models such as ChromoSSE, which model chromosome evolution and diversification rates together, we can conclude that the overall incidence of chromosome evolution in the diversification patterns of *Linum* is scarce, driving only five speciation processes in the whole phylogeny (Fig. 2). These chromosomal speciation processes involved ascending dysploidy (two speciation events), descending dysploidy (one event) and polyploidy (two speciation events). WGD events have been suggested to have occurred in the genus (Wang *et al.*, 2012; Sveinsson *et al*., 2014), suggesting that polyploidization events are not that rare in *Linum*.

There are only a few study cases that have used the ChromoSSE model so far. Freyman and Höhna (2018) were able to infer from one to 13 events of chromosomal speciation in each of six study cases. Although most of the cladogenetic events in those study cases were not explained by chromosomal speciation, the overall fit of all inferred models was better when considering one or several cladogenetic parameters of chromosomal transition (Freyman and Höhna, 2018). Recently, another study using the ChromoSSE model has shown that polyploid chromosomal speciation is highly important in the Mediterranean genus *Centaurium* Hill (Gentianaceae; Escudero *et al.*, 2023), where most of the speciation events have been linked to polyploidy. Although this model is very promising, we have to consider that all SSE models could be prone to type I error (Rabosky and Goldberg, 2015), and the ChromoSSE model may not have been an exception to this. Therefore, we urge the implementation of a ChromoSSE model that includes a hidden-state for speciation and extinction (HiSSE; Beaulieu and O’Meara, 2016), and that allows researchers to evaluate the validity of the ChromoSSE model’s findings. Regardless, it is unlikely that type I error in SSE models affected our findings, since we argue for a low incidence of chromosomal evolution on the patterns of chromosomal diversification in *Linum*.

### Chromosomal evolution and reproductive and vegetative traits

The hybrid dysfunction model of chromosomal speciation has been criticized because of the following paradox. Highly underdominant chromosomal mutations will not be established because the new cytotype is unlikely to find other individuals to mate with and will be rapidly excluded by the ancestral cytotype (i.e. the minority cytotype exclusion problem; Levin, 1975). Meanwhile, slightly underdominant chromosomal mutations will be established because the new karyotypes will be able to mate with individuals with the ancestral karyotype, but these slightly underdominant mutations will not cause speciation (White, 1978). One mechanism by which plants can overcome the minority cytotype exclusion is by self-fertilizing and therefore bypassing mate limitation; this generates the prediction of a positive relationship between self-fertilization and polyploidy. Several studies have found experimental support for this relationship (e.g. Cook and Soltis, 2000; Tate and Simpson, 2004; Barringer, 2007) but other studies do not support it (Mable, 2004). In the same direction, some studies have detected an association between polyploidization and the breakdown of heterostyly in Primulaceae and Rubiaceae (Guggisberg *et al.*, 2006; Nakamura *et al.*, 2007; Naiki, 2012). However, Naiki (2012) stated that polyploidization itself does not necessarily lead directly to the breakdown of heterostyly, so further studies are needed. In this context, the current study is the first one in which different models of chromosomal evolution have been fitted against monomorphic and polymorphic states of heterostyly, a strong driver for outcrossing (Lloyd and Webb, 1992). Here, we found no relationship between the evolution of both traits (Table 1). In fact, the distribution of chromosome number in monomorphic vs. polymorphic species overlapped completely. It is possible that the reported association between polyploidy and monomorphism is most restricted to allopolyploid lineages (Naiki, 2012), but unfortunately the frequency of allo- and autopolyploidy in *Linum* is currently unknown.

Polyploidy has traditionally been associated with perenniality (Stebbins, 1971). One possible mechanism is that the greater DNA content of perennial than annual plants increases the duration of cell cycles (Bennett, 1972). However, it has also been suggested that polyploidy is not associated with perenniality per se but rather with clonality (which is related with some forms of perenniality; Van Drunen and Husband, 2019) and/or that polyploidy is inversely related with woodiness (Rice *et al*., 2019). Our study supports a complex association between chromosome number and habit, with two different models of chromosomal evolution associated with annual vs. perennial life forms. Against expectations, our model supports higher rates of polyploidy for annual than for perennial plants (Table 2). The high rates of polyploidy detected for annual species in *Linum* (which are a minority) are explained by a few polyploidization events in terminal short branches of these species (Fig. 1). The woodiness and the probable non-clonal nature of perennial species in *Linum* (our personal observations) may explain their lower rates of polyploidy, although further studies of clonality in perennial species of *Linum* should be done to reach a solid conclusion. However, there are relatively few woody *Linum* species, which precludes further insight (Ruiz-Martín *et al.*, 2018).

### Chromosome evolution modelling meets biogeography

The important evolutionary consequences of polyploidization in plants include the potential for range expansion (Levin, 1983; Hijmans *et al*., 2007; Treier *et al*., 2009; Te Beest *et al*., 2012; Soltis *et al*., 2015). A previous study in the genus *Centaurium* (Maguilla *et al*., 2021) linking biogeographical and polyploid evolution showed that the diploid species of this genus grow in the ancestral area in the Mediterranean Basin, while polyploids have been successful in colonizing northern temperate regions (tetraploids) and southern and eastern arid regions (hexaploids). However, polyploidization does not seem to facilitate the dispersal events per se, but rather success in the new or expanded area (Maguilla *et al*., 2021). Thus, biogeographical and chromosomal evolution in *Linum* are tightly related. Our model also supports different rates of chromosomal evolution for plants in the source area vs. in colonized areas (Figs 4B and 5). Although in this case polyploidization rates are higher in the species from the source area than from colonized areas, most of these polyploid/demipolyploid events are at the tips of the phylogeny (seven events; Fig. 2). The only two events inferred in the nodes are linked to two of the four inferred colonization events, namely the origin of the South African and the South American clades. In addition, whereas the colonization event of *L. lewisii* and the North American clade is not linked to polyploidy, the origin of the South African and South American clades clearly is. Although the origin of the colonization of North America was not linked to polyploidy, four of the six species in this clade underwent polyploidy or demipolyploidy after colonization. The other two – *Sclerolinom digynum* (*n* = 6) and *L. striatum* (*n* = 9) – had three chromosome losses and chromosome stability, respectively. Interestingly, rates of dysploidy were extraordinarily high in colonized areas, which has been linked to *in situ* speciation events (Table 2; Fig. 5). In summary, almost all of the species from colonized areas (16 out of 18 in our dataset) have undergone polyploidy and/or dysploidy (Figs 1 and 2). These results seem to suggest a strong link between chromosome evolution and biogeography, as previously shown by Rice *et al*. (2015) for all angiosperms.

## SUPPLEMENTARY DATA

Supplementary data are available at *Annals of Botany* online and consist of the following.

Table S1. Studied taxa, chromosome number, stylar morphology, habit and distribution area. Figure S1. Chromosome number reconstruction based on ChromEvol ‘CONST_RATE_DEMI’ model for the dataset with all chromosome numbers. Figure S2. Chromosome number reconstruction based on ChromEvol ‘CONST_RATE_DEMI’ model for the dataset with the most probable chromosome number.

mcad139_suppl_Supplementary_Tables_S1_Figures_S1-S2
