# Supplementary material for: Drivers of diversification in Linum (Linaceae) by means of chromosome evolution: correlations with biogeography, breeding system and habit
Source: Ann Bot. 2023 Sep 20;132(5):949–62. doi: 10.1093/aob/mcad139 (PMC10808019; doi:10.1093/aob/mcad139)

Table S1. Studied taxa, chromosome number (n), stylar morphology, habit (annual vs perennial) and distribution area.

Species	Chromosome number (n)	Stylar morphology	Habit	Distribution area
Cliococca selaginoides	18	Monomorphic	Perennial	Neotropic
Hesperolinon micranthum	18	Monomorphic	Annual	Nearctic
Linum acuticarpum	15	Monomorphic	Perennial	Afrotropic
Linum africanum	15	Monomorphic	Perennial	Afrotropic
Linum album	14	Heteromorphic	Perennial	Western Palearctic
Linum alpinum	9	Heteromorphic	Perennial	Western Palearctic
Linum arboreum	14	Heteromorphic	Perennial	Western Palearctic
Linum aroanium	18	Heteromorphic	Perennial	Western Palearctic
Linum austriacum	9	Heteromorphic	Perennial	Western Palearctic
Linum austriacum subsp. *mauritanicum*	9	Heteromorphic	Perennial	Western Palearctic
Linum bienne	15	Monomorphic	Annual, biennial	Western Palearctic
Linum campanulatum	14	Heteromorphic	Perennial	Western Palearctic
Linum capitatum	14	Heteromorphic	Perennial	Western Palearctic
Linum catharticum	8	Monomorphic	Annual	Western Palearctic
Linum comptonii	15	Heteromorphic	Perennial	Afrotropic
Linum corymbiferum	9	Heteromorphic	Perennial, biennial	Western Palearctic
Linum corymbulosum	9	Monomorphic	Annual	Western Palearctic
Linum decumbens	9	Monomorphic	Annual, biennial	Western Palearctic
Linum elegans	14	Heteromorphic	Perennial	Western Palearctic
Linum flavum	15	Heteromorphic	Perennial	Western Palearctic
Linum gallicum	10	Monomorphic	Annual	Western Palearctic
Linum gracile	15	Monomorphic	Perennial	Afrotropic
Linum grandiflorum	8	Heteromorphic	Annual	Western Palearctic
Linum gyaricum	15	Heteromorphic	Perennial	Western Palearctic
Linum heterostylum	15	Heteromorphic	Perennial	Afrotropic
Linum hirsutum	8	Heteromorphic	Perennial	Western Palearctic
Linum hologynum	21	Monomorphic	Perennial	Western Palearctic
Linum kingii	13	Monomorphic	Perennial, biennial	Nearctic
Linum leonii	9	Monomorphic	Perennial	Western Palearctic
Linum lewisii	9	Monomorphic	Perennial	Nearctic
Linum littorale	36	Monomorphic	Perennial	Neotropic

Linum macraei	36	Monomorphic	Perennial	Neotropic
Linum maritimum	10	Heteromorphic	Perennial	Western Palearctic
Linum mucronatum	14	Heteromorphic	Perennial	Western Palearctic
Linum narbonense	14	Heteromorphic	Perennial	Western Palearctic
Linum nervosum	15	Heteromorphic	Perennial	Western Palearctic
Linum nodiflorum	13	Monomorphic	Annual	Western Palearctic
Linum pallescens	9	Monomorphic	Biennial, perennial	Eastern Palearctic
Linum perenne	9	Heteromorphic	Perennial	Western Palearctic, Eastern Palearctic
Linum prostratum	18	Monomorphic	Perennial, annual	Neotropic
Linum pubescens	8	Heteromorphic	Annual	Western Palearctic
Linum punctatum	9	Heteromorphic	Perennial	Western Palearctic
Linum pycnophyllum	9	Heteromorphic	Perennial	Western Palearctic
Linum rupestre	18	Monomorphic	Perennial	Nearctic
Linum seljukorum	8	Monomorphic	Annual	Western Palearctic
Linum setaceum	9	Monomorphic	Annual	Western Palearctic
Linum stelleroides	9	Monomorphic	Annual, biennial	Eastern Palearctic
Linum striatum	9	Monomorphic	Perennial	Nearctic
Linum strictum	9	Monomorphic	Annual	Western Palearctic
Linum suffruticosum	36	Heteromorphic	Perennial	Western Palearctic
Linum tauricum	15	Heteromorphic	Perennial	Western Palearctic
Linum tenue	10	Heteromorphic	Annual	Western Palearctic
Linum tenuifolium	9	Monomorphic	Perennial	Western Palearctic
Linum trigynum	10	Monomorphic	Annual	Western Palearctic
Linum usitatissimum	15	Monomorphic	Annual	Western Palearctic
Linum vernale	15	Monomorphic	Annual	Nearctic
Linum viscosum	8	Heteromorphic	Perennial	Western Palearctic
Radiola linoides	9	Monomorphic	Annual	Western Palearctic, Afrotropic
Reinwardtia indica	11	Heteromorphic	Perennial	Eastern Palearctic
Sclerolinon digynum	6	Monomorphic	Annual	Nearctic

Figure S1. Chromosome number reconstruction based on ChromEvol CONST_RATE_DEMI for the dataset with all chromosome number. Chromosome numbers and probabilities (in plot charts) are shown with different colors.

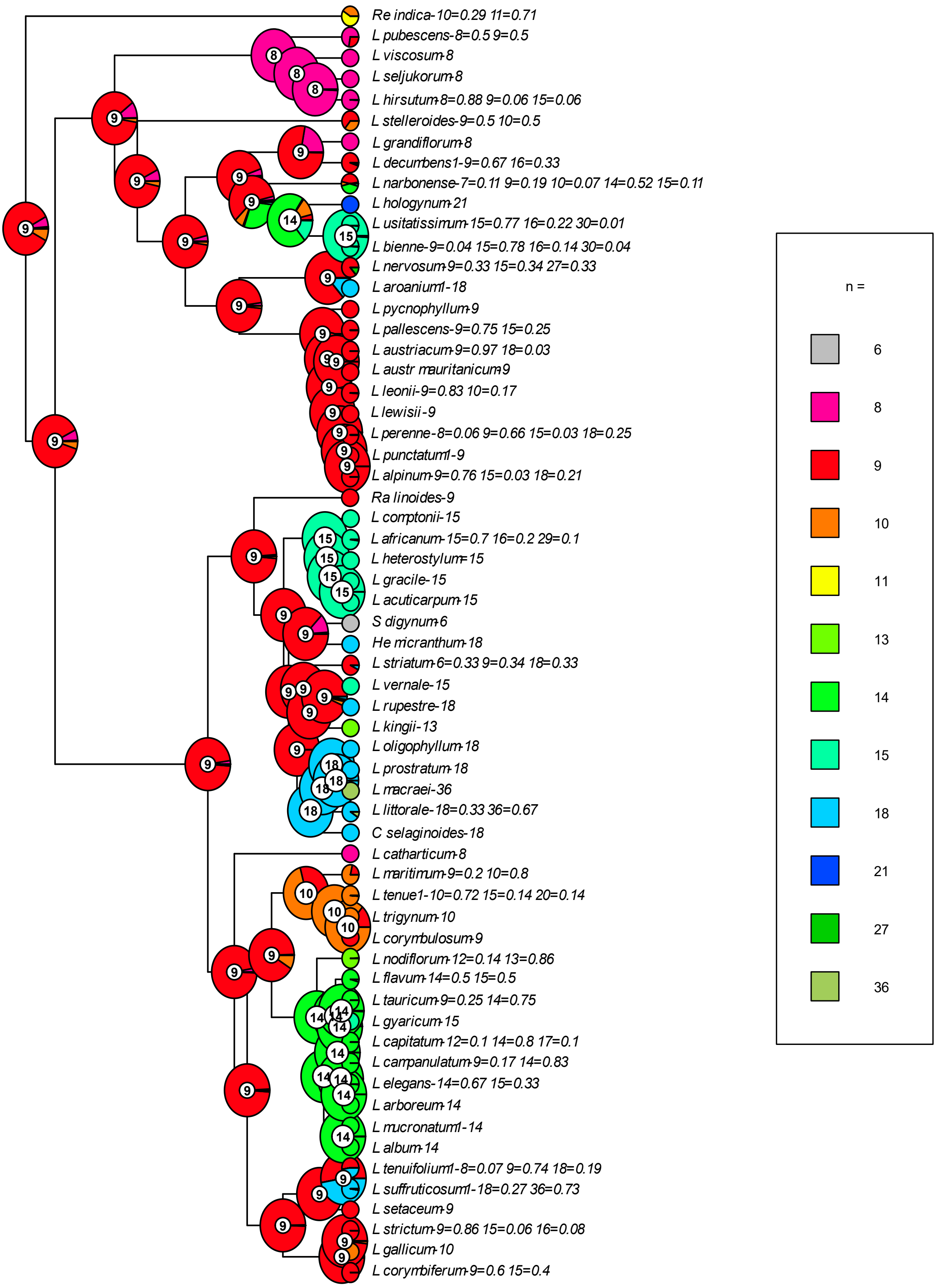

Figure S2. Chromosome number reconstruction based on ChromEvol CONST_RATE_DEMI for the dataset with the most probable chromosome number. Chromosome numbers and probabilities (in plot charts) are shown with different colors.

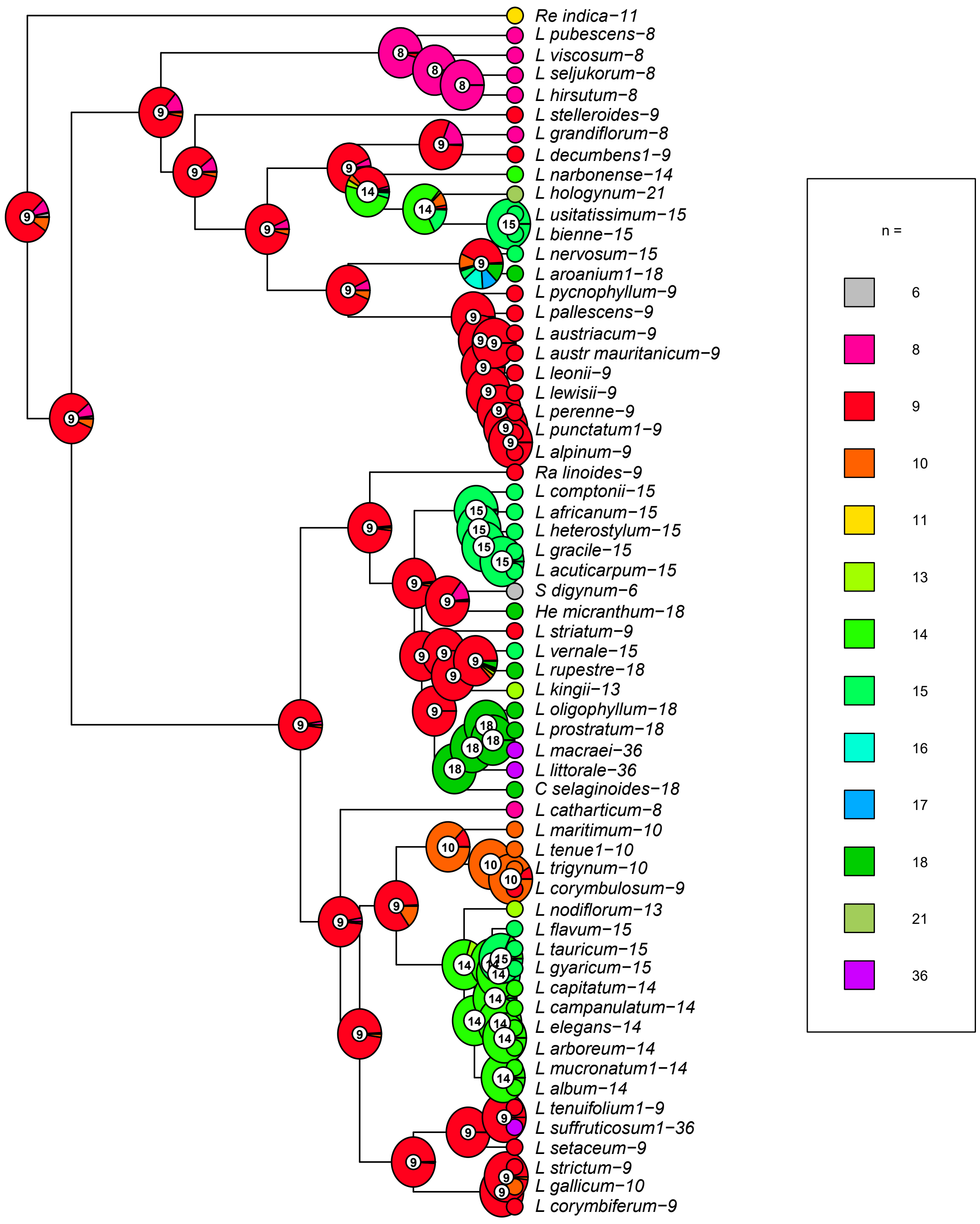

Supplement: mcad139_suppl_Supplementary_Tables_S1_Figures_S1-S2 [file mcad139_suppl_supplementary_tables_s1_figures_s1-s2.pdf]
